# Tetramethylpyrazine Ameliorates Lipopolysaccharide-Induced Sepsis in Rats *via* Protecting Blood–Brain Barrier, Impairing Inflammation and Nitrous Oxide Systems

**DOI:** 10.3389/fphar.2020.562084

**Published:** 2020-10-02

**Authors:** Zi-Sheng Huang, Da-Qi Xie, Li-Jun Xu, Chang-Shun Huang, Min Zheng, Yi-Jun Chen, Yin Cao

**Affiliations:** ^1^Department of Anesthesiology, Ningbo First Hospital, Ningbo, China; ^2^Department of Cardiology, Ningbo Ninth Hospital, Ningbo, China; ^3^Ophthalmology Operating Room, Ningbo First Hospital, Ningbo, China; ^4^Department of Anesthesiology, Ningbo Ninth Hospital, Ningbo, China

**Keywords:** tetramethylpyrazine, lipopolysaccharide, inflammation, blood–brain barrier, nitrous oxide system

## Abstract

The aim of this study was to assess the underlying impact of Tetramethylpyrazine (TMP), which is the main activity compound of *Ligusticum chuanxiong* Hort, on the blood–brain barrier, inflammatory and nitrous oxide systems in a rat model of lipopolysaccharide (LPS)-induced sepsis. The SD rats were divided into control group, LPS treatment group, and LPS + TMP treatment group. TMP administered by tail vein injection. The mortality of experimental rats was recorded during the experiment. Rats were sacrificed after 14 days. Peripheral blood was collected and the expression levels of inflammatory factors TNF-α, IL-1β, and IL-6 were detected by ELISA. The integrity of blood-brain barrier was detected by sodium fluorescein staining. Lung and brain tissues were taken to detect the infiltration of immune cells. Immunohistochemistry was performed to detect the expression of tight junctions related proteins and oxidative stress-related proteins. The results showed that TMP treatment for 14 days significantly decreased the weight loss and increased the survival rate of the septic rats significantly. TMP decreased the infiltration of inflammatory cells and alleviated the sepsis-induced damage in both the lung and brain tissues. The inflammatory cytokines TNF-α, IL-1β, and IL-6, were significantly decreased post-TMP treatment. Histopathological analysis with sodium fluorescein staining density showed that TMP had a protective effect on the basal lamina and cerebral cortex. Also, TMP significantly increased expression of the tight junction-related proteins claudin-5 and occludin in the brain tissue and increased the expression of the *ZO-1*, *Occludin*, and *Claudin-5* genes, indicating alleviated the degree of blood–brain barrier destruction. Furthermore, immunohistochemistry (IHC) and immunoblotting confirmed that TMP could inhibit the indicators of the nitrous oxide system, iNOS and eNOS; in addition, TMP significantly decreased the levels of MDA and NO. The findings showed that TMP treatment during sepsis was associated with the protection of the blood–brain barrier and the suppression of inflammatory reactions and the nitrous oxide system. This study reveals a promising protective role of TMP in septic encephalopathy and may suggest a therapeutic approach for fighting the deadly disease of sepsis in the clinic.

## Introduction

Sepsis, a systemic inflammatory response syndrome (SIRS), is caused by infection due to the presence of bacteria (Sursal et al., 2013). As reported, sepsis is mainly associated with bacterial lipopolysaccharides (LPS) and is also called endotoxemia (Lykkesfeldt et al., 2014; Xu et al., 2018). As a severe problem in the field of critical illness, sepsis is increasingly attracting the attention of clinicians and researchers.

In the past decade, the rapid development of modern biotechnology has dramatically promoted the public understanding of sepsis pathogenesis and the improvement of sepsis treatment (Harisha, 2014). According to a large amount of clinical and experimental research, the data on sepsis treatment and new drug development did not seem to be promising (Fuchs et al., 2019; Zhang et al., 2019). Strategies for intervention in sepsis have shown useful application prospects in animal experiments; however, they have not achieved significant clinical efficacy in phase II and phase III clinical trials, and some have even exacerbated the disease (Schimmer et al., 2016; Łysenko et al., 2017). Thus far, the mortality rate of severe sepsis or septic shock is still approximately 50%. The critical factors causing the unfortunate effects of these agents may be the choices of improper animal models for the preclinical animal experiments. As reported, bacterial infection or bacterial LPS can be used to induce sepsis animal models (Sterns et al., 2005; Xu et al., 2018). In our previous study, LPS injection was shown to be an ideal and consistent protocol for establishing a sepsis model.

Tetramethylpyrazine (TMP), also named 2, 3, 5, 6-tetramethylpyrazine hydrochloride, is the main active ingredient in the traditional Chinese medicine Chuanxiong (*Ligusticum chuanxiong* Hort., Umbelliferae) (Juan et al., 2007). TMP is currently used in the treatment of cardiovascular and cerebrovascular diseases (Guo et al., 1983; Juan et al., 2007). Several studies have confirmed that TMP has an inhibitory effect on sepsis by regulating oxidative stress and ischemic hypoxia (Lin et al., 2009; Yingke et al., 2016; Liu et al., 2018). A systemic inflammatory response accompanied by the release of free radicals is often considered to be the underlying mechanism of sepsis in individuals. These free radicals cause the infiltration of various inflammatory cells, stimulate the release of cytokines, such as tumor necrosis factor and interleukins, and enhance the peroxidation of lipid *in vivo* (Lin et al., 2009; Dawé et al., 2018). Studies have shown that TMP can effectively remove oxygen free radicals and reduce tissue damage. The change in the permeability of the blood–brain barrier is highly associated with oxidative damage and vasodilation; thus, the removal of free radicals and vasodilation may be essential in the treatment of sepsis (Lin et al., 2009; Geven et al., 2018).

Previous studies have shown that TMP can inhibit lipopolysaccharide (LPS)-induced endothelial nitric oxide synthase in rat astrocytes, and its mechanism is related to NOS. The present study aimed to investigate the effects of TMP on LPS-induced sepsis in the sepsis rat model and to explore the underlying mechanism related to the blood–brain barrier, inflammation, and nitrous oxide systems.

## Materials and Methods

### Animals

The SPF male SD rats weighing 200-250 g were provided by Shanghai Jiesijie Experimental Animal Co., Ltd. The production license number is SCXK (Shanghai) 2018-0004. The temperature of the breeding room was 20–25°C, and the relative humidity was 40–70%. The animal experiment protocol of this study has been approved by the Animal Experimentation Ethics Committee of Ningbo First Hospital.

### Establishment of the LPS-Induced Sepsis Model

The rats were randomly divided into the control, LPS (model) and LPS+TMP groups. The LPS and LPS+TMP groups were injected with a dose of 5 mg/kg LPS (Cat: L1880, Solarbio, Beijing, China) through the tail vein (Lin et al., 2009; Liu et al., 2018). After half an hour, the rats were debilitated, moved slowly and had diarrhea, indicating that LPS toxicity had begun to affect the whole bodies of the rats. Then, the LPS+TMP group was injected with 10 mg/ml TMP (Sigma-Aldrich, St. Louis, MO, USA) through the tail vein for treatment. Another 8 rats were injected with normal saline as the control group.

### Body Weight and Survival Rate

Another 24 SD rats were randomly divided into 3 groups: the control group, the LPS group and the LPS+TMP group, with 8 rats in each group. After the sepsis model was established, the body weight was recorded each day, and the survival rate was calculated. The treatment lasted for 14 days.

### Bronchoalveolar Lavage Fluid Collection and Treatment

After LPS infusion for 24 h, rat was sacrificed and its trachea was exposed. A sterile 18 G trocar was inserted into the trachea and sutured with silk thread. The lung was instilled and withdraw with sterile PBS (10 ml per time) for three times. The bronchoalveolar lavage fluid (BALF) was collected and samples were centrifuged at 2,000 g at 4°C for 10 min. The supernatant is collected and aliquoted, stored at −80°C for subsequent testing.

### Histopathological Observation of the Brain and Lung Tissues

The rats were injected with an excessive amount of 10% chloral hydrate, and the abdominal cavity was cut along the midline of the abdomen. The fixed brains were embedded in paraffin. After cardiac perfusion to remove the blood from the blood vessels, the skull was carefully opened, and the entire brain was removed. After sectioning of the brain tissue, HE staining was used for the morphological observation of the brain tissues, especially the hippocampus, which is responsible for cognitive function. The lung tissue was perfused and washed with 100 mL of physiological saline and then fixed in a formaldehyde solution for 24 h for pathological examination. The HE staining steps were as follows: the slides were immersed in xylene (I) for 15 min, xylene (II) for 15 min, xylene: absolute ethanol = 1:1 for 2 min, 100% ethanol (I) for 5 min, 100% ethanol (II) for 5 min, 80% ethanol for 5 min, distilled water for 5 min, and a hematoxylin staining solution for 5 min. The slides were then washed with water for 10 min or with running water for 5 min, followed by fading with 1% hydrochloric acid ethanol for 30 s. The slides were washed with water for 30 s, washed with distilled water for 5 s, and stained with a 0.5% eosin solution for 1–3 min. Then, the slides were gently washed with distilled water for 30 s, gently washed with 80% ethanol for 30 s, and immersed in 95% ethanol (I) for 1 min, 95% ethanol (II) for 1 min, absolute ethanol (I) for 3 min, xylene (I) for 3 min, and xylene (II) for 3 min. The slides were sealed with neutral gum after being dried.

The pathological lesions of the brain and lung tissue were observed. The amount of inflammatory infiltration and whether the inflammatory model had been successfully established were determined using optical microscopy.

### The Detection of Serum Indicators by ELISA

After the rats were anesthetized, the abdomen was opened, and the abdominal aortic blood was collected into an anticoagulant tube containing 1% heparin sodium, centrifuged, the supernatant was separated, and aliquoted and stored at −80°C. At the same time, the lung tissue of each rat was taken with a weight ratio of 1:9 with PBS to prepare a tissue homogenate, and the supernatant was centrifuged at 2,000g for ELISA analysis. The expression levels of IL-6 (Cat : EK306/3-96, Multisciences Biotech, Hangzhou, China), TNF-α (Cat : EK382/3-96, Multisciences Biotech, Hangzhou, China), and IL-1β (Cat : EK301B/3-24, Multisciences Biotech, Hangzhou, China) in the serum and lung tissue homogenate of the normal control group, LPS group, and LPS+TMP group were detected by ELISA. According to the ELISA kit instructions, the serum indicators were then measured using a microplate reader (Molecular Devices), and results were analyzed.

### Immunohistochemistry (IHC) of Brain Tissue

Paraffin-embedded sections of brain tissues containing the hippocampus were used for IHC. The expression and distribution of the occluding (Cat: A2601, ABclonal, Wuhan, China), claudin-1(Cat: RT1141, Huabio, Hangzhou, China), and claudin-5 (Cat: A10207, ABclonal, Wuhan, China) proteins were detected. First, the paraffin-embedded sections were heated at 60°C for 15 min to melt the wax and dry, and then, the sections were deparaffinized and rehydrated. The sections were placed in a pH 6.0 citrate buffer solution and incubated at 121°C for 15 min. The sections were washed 3 times with PBS buffer, and liquid flow prevention control was used on the section border. The tissues were incubated in a 3% hydrogen peroxide solution at room temperature for 10 min and blocked in a 1% BSA solution for 15 min. Then, the tissues were incubated with the primary antibody (1:100 dilution) at 4°C overnight. Next, the tissues were incubated with the HRP-conjugated secondary antibody (Cat: HA1001, Huabio, Hangzhou, China) (1:100 dilution) at room temperature for 1 h and washed 3 times with TBST buffer. After rinsing with water, the sections were stained with a DAB solution for minutes. The slides were counterstained with hematoxylin for 30 s followed by rinsing in running water for 1 min. The sections were blued in 0.1% sodium bicarbonate for 1 min. The slides were sealed with neutral gum after air drying. The IHC images were observed and taken under a microscope.

### The Detection of Blood–Brain Barrier Damage

Twenty-three hours after the establishment of the sepsis model, 2 rats were randomly selected from each group and injected with a 3 mg/kg sodium fluorescein solution (Cat.F8140, Solarbio, Beijing, China), which can be detected in the rat’s urine half an hour after injection. Then, the rats were sacrificed, and their tissues were sampled after one hour. Transverse sections of the same site of the brains of the rats injected with sodium fluorescein were placed on clean glass slides after 6 h of fixation. Images of the brain sodium fluorescein staining were taken with a gel imager. The gray value was measured and quantified by ImageJ software.

### Real-Time PCR Detection

Partial brains were used to quantify the expression of *ZO-1*, *occludin*, and *claudin-5* by real-time PCR. The primers for the *ZO-1*, *Occludin*, and *Claudin-5* genes were designed through the NCBI website (https://www.ncbi.nlm.nih.gov/). β-actin served as an internal control. After verifying the primers and determining the specificity of the primers, TRIzol was used to extract the total RNA. After detecting the RNA concentration and analyzing the RNA by gel electrophoresis, the obtained RNA was determined to be complete. The cDNA was obtained by reverse transcription with a reverse transcription kit, and then real-time PCR was performed using a real-time PCR kit on 7500 fast real-time PCR(ABI). The amplification conditions were as follows: predenaturation, 95°C for 2 min; PCR, 40 cycles of 95°C for 20 s, 55°C for 30 s, 72°C for 40 s. The fluorescence signal was detected at 72°C, and the relative expression of the genes was calculated based on the *Ct* value. The primer sequences are shown in **Table 1**.

**Table 1:** Primer sequences for RT-PCR.

Gene	Primer sequence	
OCLN	Forward	5’CCCTTCTTTCCTTAGGCGACC3’
	Reverse	5’TGGGTTTGAATTCATCCGGC3’
ZO-1	Forward	5’ GATAGCCCTGCAGCCAAAGA3’
	Reverse	5’ ACAATGCGGCGATAAACGTC 3’
Claudin5	Forward	5’ CTGCCTTAATGTCCGGTGGT 3’
	Reverse	5’ AAACCCCAAGCTTCGAGGAG 3’
β-actin	Forward	5’ TGAGAGGGAAATCGTGCGTGAC 3’
	Reverse	5’ GAACCGCTCGTTGCCAATAGTG 3’

### Western Blot

Brain tissue was taken, the tissue was lysed on ice with RIPA lysis buffer, and the lysed material was blown with 1 mL pipette to destroy the DNA. After centrifuge, 3μL supernant was collected for BCA assay to quantify, and the remaining samples were incubated at 95°C for 5–10 min for denaturation and stored at −20°C. Prepare 10% SDS-PAGE gel, add 40 g protein per well, after electrophoresis separation of protein, 200 mA constant current for 1 hour to transfer protein to PVDF membrane, then blocked with 5% skim milk for 1 hour at room temperate. Then the primary antibody was incubated overnight at 4°C,including ZO-1, claudin-1, claudin-5, Occludin, eNOS were all purchased from ABclonal (Wuhan, China), iNOS (Cat:189885-1-AP, Proteintech, USA) and the internal reference antibody β-actin (Cat:R1102-1, Huabio, Hangzhou, China). Then membrane was washed with TBST for three times, and incubated with secondary antibody at room temperature for 1 h, and then membrane was washed with TBST for three times. TBST was discarded, ECL luminescent solution was added, and developed in the protein imager. Image J was used to quantify the gray value of the band.

### The Detection of NOS-Related Indicators

The serum samples of each group were collected, in which the level of NO (Cat: S0021, Beyotime, Shanghai, China) and MDA (Cat: S0131S, Beyotime, Shanghai, China) was determined to follow the kit instruction. Paraffin-embedded sections were used for IHC staining to detect the *eNOS* and *iNOS* expression patterns in the brain. The sections were observed, and the images were taken under a microscope. Image J was used for quantitative analysis.

### Statistical Analysis

The data were analyzed using SPSS 21.0 software. The statistical significance analysis was performed by analysis of variance (one-way ANOVA) for multiple group comparisons or *t*-tests for two group comparisons. Differences were considered significant at the *P*<0.05 or *P*<0.01 level.

## Results

### TMP Reduced Body Weight Changes and Improved Survival Rates

**Figure 1:**
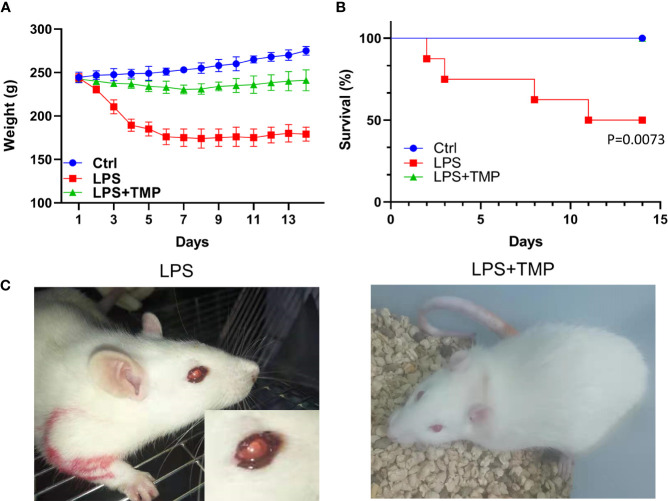
Weight change and survival rate of rats with LPS-induced sepsis. **(A)** The trends of weight changes of rats in the control group, LPS group and LPS+TMP group for 14 days; **(B)** The survival rates of each group for 14 days. **(C)** Modeling and enlarged eye detail of rats in the LPS and LPS+TMP groups 4 h after injection with LPS.

The bodyweight of the LPS group decreased sharply in the first four days. After LPS administration, the rats injected with TMP at a dose of 10 mg/kg showed a significant improvement in their symptoms, and the effects of LPS on the body weights were relatively consistently reduced. Based on calculations of the survival rate of the rats, the mortality of the LPS group significantly increased within 14 days (*P˂*0.01), reaching 50%, while the rats injected with TMP did not die within 14 days, which may indicate that TMP can rescue LPS-induced sepsis in rats to some extent and that TMP has an evident protective effect on sepsis. These data are shown in **Figures 1A, B**.

After the LPS-induced sepsis model was established, morphological observations of the rats were conducted within half an hour after the injection of 1 mg LPS, and the results showed that the activity of the rats was significantly reduced. One hour after establishing the model, symptoms, such as diarrhea, appeared. After 4 h, bleeding occurred in the eyes of the rats (**Figure 1C**).

### TMP Ameliorates Lung and Brain Pathological Damage

**Figure 2:**
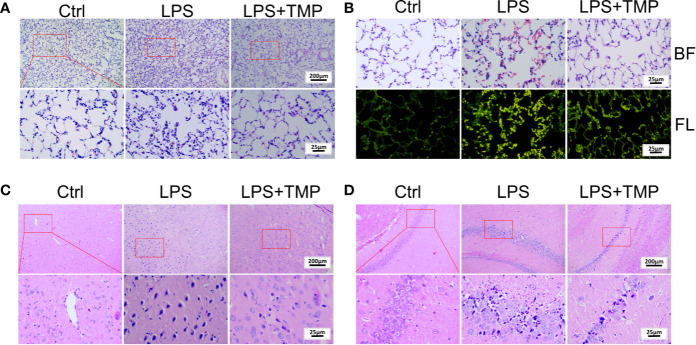
Pathological observation of lung and brain tissue by HE staining. **(A)** Lung tissue (Upper, 100×; Lower, 400×). **(B)** Lung tissue observed under a fluorescence microscope (400×). BF: Brightfield; FL: Fluorescence. **(C)** Cerebral cortex (Upper, 100×; Lower, 400×). **(D)** Hippocampus (Upper, 100×; Lower, 400×).

The HE staining results are shown in **Figure 2A**. There was no histological damage in the lung tissue of the control group. In the LPS group, the inflammatory cell infiltration into the alveolar cavity increased, the alveolar space was thickened, and the alveolar cavity contained more inflammatory exudations. Additionally, some alveoli atrophied or ruptured, a large number of red blood cells and protein solution exuded in the alveoli, and a large number of neutrophils aggregated in the interval. In the LPS+TMP group, there was only a slight inflammatory cell infiltration, edema was absent in the alveolar space absent, and no obvious exudate was observed in the alveolar cavity. Moreover, the alveolar structure was complete. In addition, observation of HE-stained sections of the lung tissue under fluorescence microscopy showed that the amount of bleeding in the lungs of the rats in the LPS group was significantly higher than that in the lungs of the rats in the control group and the LPS + TMP group (**Figures 2B****)**.

Moreover, HE staining of the brain tissue showed that a large number of neuronal cells in the cerebral cortex of the LPS group atrophied, and the nuclei were deeply stained, indicating that LPS caused damage to rat neuronal cells. By comparison, these symptoms were significantly reduced in the LPS + TMP group (**Figure 2C**). Then, HE staining of the hippocampus was performed in each group of rats (**Figure 2D**). LPS also damaged the neurons in the hippocampus. The neurons were more condensed, the nuclei were deeply stained, and the cells were loosely arranged. The phenomenon corresponded to the external behaviors of the rats, such as staggering and looking around. Injection with TMP reduced the LPS-induced damage to the rat neural cells.

### Effect of TMP on Serum and Lung Inflammatory Cytokines in Septic Rats

**Figure 3:**
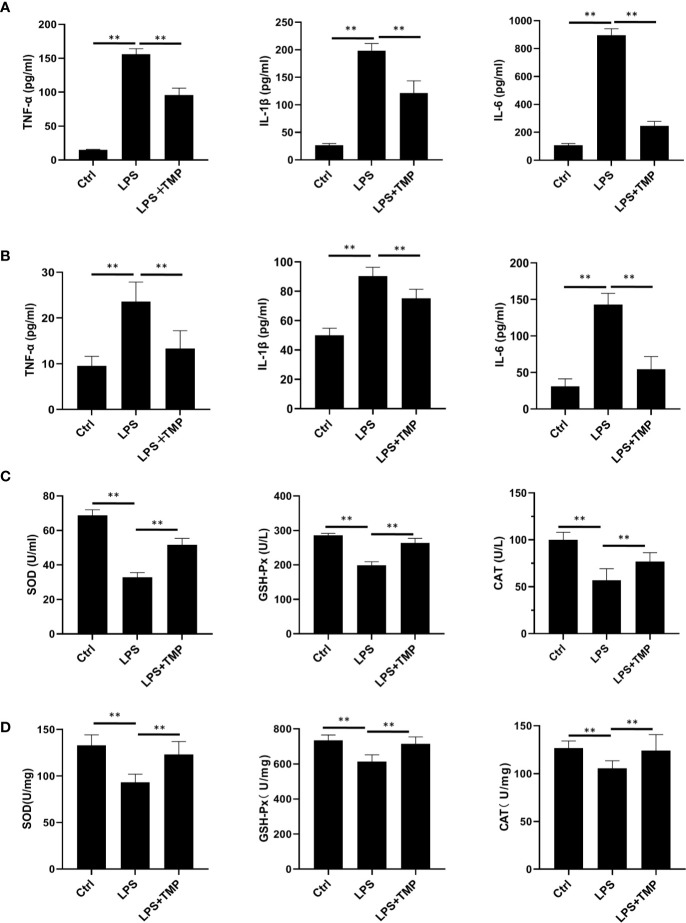
Effect of TMP on the inflammatory cytokines and antioxidant enzymes in the serum of septic rats (*n*=8). **(A)** The expression level of inflammatory factors in serum. **(B)** The Expression levels of inflammatory cytokines in rat lung lavage fluid. **(C)** The expression levels of antioxidant enzymes in Serum. **(D)** The expression levels of antioxidant enzymes in lung tissue. Compared with the LPS group, ^**^P < 0.01.

The ELISA results showed that the serum (**Figure 3A**) and lung lavage fluid (**Figure 3B**) levels of IL-6, IL-1β, and TNF-α were all increased in the LPS group, and these levels were significantly different when compared with the levels in the control group (*P <*0.01). The levels of the inflammatory cytokines IL-6, IL-1β, and TNF-α in the LPS+TMP group were significantly decreased (*P*<0.01). It is worth mentioning that the levels of TNF-α and IL-1β in the LPS+TMP group increased to levels higher than those in the control group, while IL-6 decreased (*P <*0.01). The oxidative stress indexes in blood (**Figure 3C**) and lung tissues (**Figure 3D**) were detected. The results showed that SOD (Cat: S0109, Beyotime, Shanghai, China), GSH-PX(Cat: S0056, Beyotime, Shanghai, China) and CAT(Cat: S0051, Beyotime, Shanghai, China) indexes in blood and lung homogenate of LPS group were significantly reduced, and there were significant differences between them (*P*<0.01). After TMP treatment, the indicators related to oxidative stress were significantly improved compared with the LPS group (*P*<0.01).

### The Protective Effect of TMP on the LPS-Induced Blood–Brain Barrier Destruction in Sepsis

**Figure 4:**
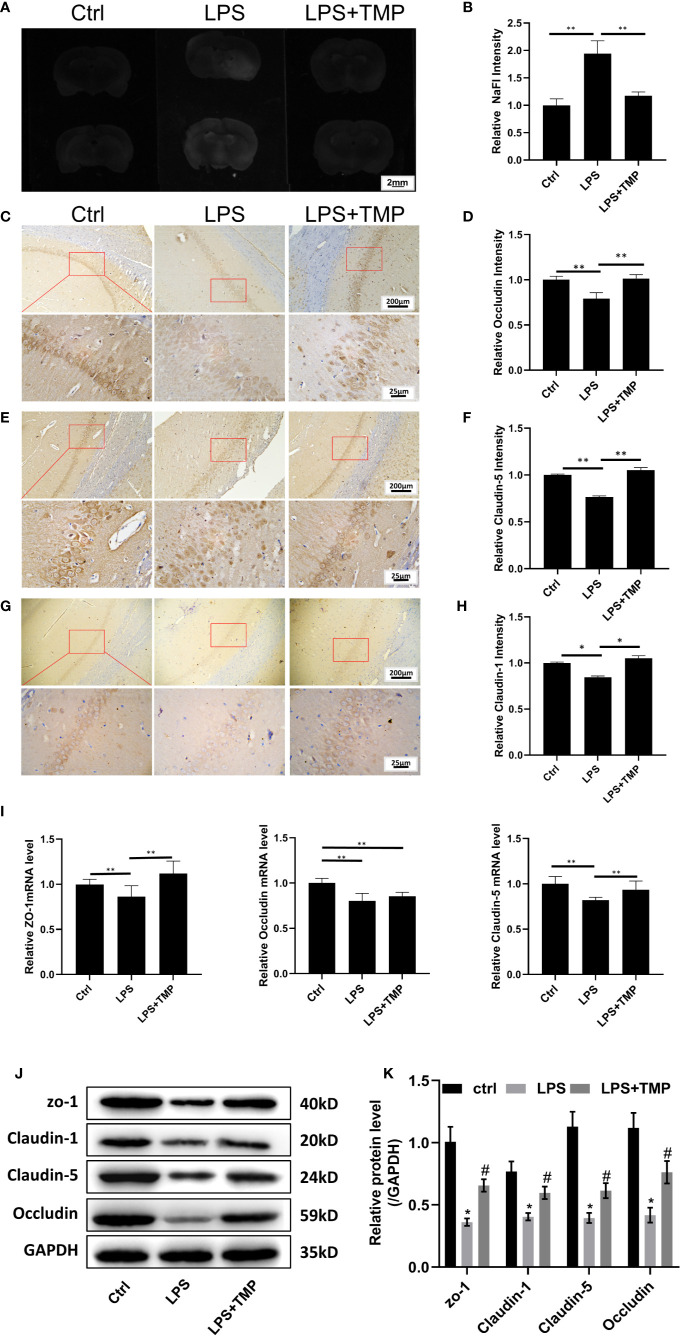
TMP has a protective effect on the LPS-induced BBB destruction in sepsis. **(A)** Sodium fluorescein staining revealed the degree of blood–brain barrier damage, and the images were taken by a UV gel imager. **(B)** The relative intensity of the Sodium fluorescein signaling is calculated. **(C)** IHC staining of the occludin in hippocampus of the rats (Upper, 100×; Lower, 400×). **(D)** The relative IHC intensity of occludin protein; **(E)** IHC staining of the Claudin-5 in hippocampus of the rats (Upper, 100×; Lower, 400×). **(F)** The relative IHC intensity of Claudin-5 protein; **(G)** IHC staining of the Claudin-1 in hippocampus of the rats (Upper, 100×; Lower, 400×). **(H)** The relative IHC intensity of Claudin-1 protein; **(I)** The relative levels of the *ZO-1*, O*ccludin*, and *Claudin-5* genes in the brains of each group (*n*=8). **(J–K)** Western blot results and Histogram shows statistical quantified results. Compared with the LPS group, ^*^P < 0.05, ^**^P < 0.01. Compared with the Control group, ^#^P < 0.05.

The results showed that sodium fluorescein stained the brains from the basal lamina and cerebral cortex. Compared with that in the control group and LPS+TMP group, fluorescence staining was more evident in the LPS group, and the difference was significant (*P*<0.01). However, the difference between the LPS+TMP group and the control group was not significant (*P >*0.05) (**Figures 4A, B****)**.

The IHC staining results of *Occludin*, *Claudin-5* and *Claudin-1* are shown in **Figures 4C, E, G**, respectively. Compared with that in the control group, the expression of the *Occludin*, *Claudin-1* and *Claudin-5* proteins in the hippocampus in the LPS group was decreased, and the differences were significant (*P*<0.01). Compared with that in the LPS+TMP group, the expression of the *Occludin*, *Claudin-1* and *Claudin-5* proteins in the hippocampus in the LPS group was decreased, and the differences were significant (*P <*0.01). Comparing the LPS+TMP group with the control group, the expression levels of the *Occludin*, *Claudin-1* and *Claudin-5* proteins in the hippocampus of the brain were almost the same, and the differences were not significant (*P*>0.05).The image was grayed and quantified by ImageJ software (**Figures 4D, F, H****)**.

The relative expression of the *ZO-1*, *occluding*, and *claudin-5* genes in the brains of each group was detected. Comparing the LPS group with the control group, the expression levels of *ZO-1*, *occludin*, and *claudin-5* in the brains of the rats treated with LPS were all reduced, and the differences were significant (*P <*0.01). Comparing the LPS group with the LPS+TMP group, the relative expression levels of the *ZO-1* and *Claudin-5* genes in the brains of the LPS + TMP group were increased, and the differences were significant (*P <*0.01). However, the difference in *occludin* gene expression between the two groups was not significant (*P >*0.05). Comparing the control group with the LPS+ TMP group, the expression of the *ZO-1* gene was increased in the LPS+TMP group, and the difference was significant (*P <*0.01). *Occludin* expression was lower in the brains of the LPS+TMP group than that in the brains of the control group, and this difference was significant (*P*<0.01), while *claudin-5* gene expression was not significantly different between the LPS+TMP group and the control group (*P*>0.05) (**Figure 4I**).

Then, protein in rat brain was extracted, and the western blot was performed, the results show that tight junction proteins in LPS group, including *ZO-1*, *Claudin-1*, *Claudin-5*, and *Occludin* are all decreased, there was a significant difference between the LPS group and control group, while TMP treatment could effectively improve the tight junction associated-protein expression level, which show significant differences with LPS group (**Figure 4J**).

### TMP Suppressed the Expression of Nitric Oxide Synthase in Septic Rats

**Figure 5:**
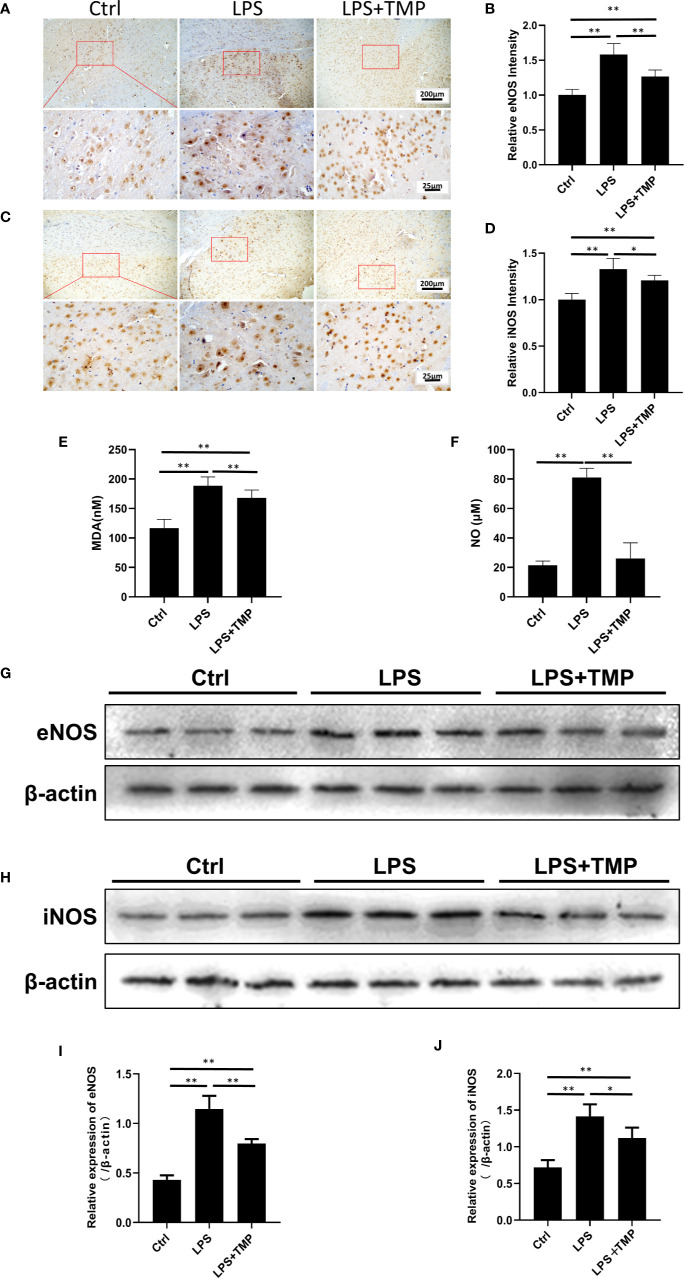
Effects of TMP on NOS and serum MDA and NO. **(A)** IHC staining of the eNOS in cerebral cortex of the rats (Upper, 100×; Lower, 400×). **(B)** The relative IHC intensity of eNOS protein; **(C)** IHC staining of the iNOS in cerebral cortex of the rats (Upper, 100×; Lower, 400×). **(D)** The relative IHC intensity of iNOS protein; **(E, F)** Serum MDA and NO contents in the control group, LPS group and LPS+TMP group (*n*=8). **(G–J)** Western blot was used to detect the expression levels of eNOS and iNOS in rat cerebral cortex and histogram shown the quantified results. Compared with the LPS group, ^*^P < 0.05, ^**^P < 0.01.

The expression and location of *eNOS* and *iNOS* in the brain were clearly shown by IHC (**Figures 5A, C****)**. The image was grayed and quantified by ImageJ software (**Figures 5B, D****)**. Compared with that in the control group, the expression of the *eNOS* and *iNOS* proteins in the cerebral cortex in the LPS group was increased, and the differences were significant (*P <*0.01). Compared with those in the LPS group, the expression levels of the *eNOS* and *iNOS* proteins in the cerebral cortex in the LPS+ TMP group were decreased, and the differences were statistically significant (*P*<0.05). **Figures 5I, J** were the quantitative results of eNOS and iNOS by Western blot.

As shown in **Figure 5E**, compared with that in the control group, the MDA content in the LPS and LPS+TMP groups was significantly increased. The rats in both groups had lipid oxidative damage, but the difference was significant in the LPS+TMP group (*P*<0.01). Compared with that in the LPS group, the MDA content in the LPS+TMP group was decreased, and the difference was statistically significant (*P*<0.01). The NO test results (**Figure 5F**) showed that the serum NO content in the LPS group significantly increased (*P*<0.01), while the difference between the LPS+TMP group and the control group was not significant (*P*˃0.05). Furthermore, the expression levels of *eNOS* and *iNOS* of rat cortical tissue were detected by immunoblotting (**Figure 5G**), in compared with the control group, the expressions of *eNOS* and *iNOS* in the LPS group were significantly up-regulated (*P <*0.05), while the expressions of *eNOS* and *iNOS* induced by LPS can be attenuated in TMP group (*P*<0.05), and the WB results are quantified (**Figure 5H**). The above results suggest that TMP could downregulate the NO level in rat brain tissue mostly by inhibiting the expression of *eNOS* and *iNOS* induced by LPS.

## Discussion

Sepsis occurs in approximately 2% of hospitalized patients and 75% of intensive care unit (ICU) patients, and sepsis has a mortality rate of 20–50%. Clinically, the mortality of sepsis patients with delirium, shock and other symptoms is higher. In the past few years, observation methods have been used in most cell physiology studies (Andrikopoulos et al.; Dal-Pizzol et al., 2014; Kuperberg and Wadgaonkar, 2017; Qiang et al., 2019). To better understand the occurrence, development and other pathological changes in the inflammatory reactions that occur *in vivo* during sepsis, clinically relevant animal models of sepsis or septic shock are needed (Andrikopoulos et al.; Stare et al., 2015; Arslan et al., 2017). In addition, study has shown that estrogen in female mice can inhibit the occurrence of sepsis by inhibiting platelet activation, indicating that the occurrence of sepsis could be affected by gender (Xudong et al., 2015).

It is well known that lipopolysaccharide (LPS, endotoxin) in the cell wall of Gram-negative bacteria is an effective trigger for the release of host-derived inflammatory mediators. Moreover, a large number of experiments have proven that most of the sepsis-induced shock and death in humans are caused by the sustained action of acute inflammation (Toscano et al., 2011; Elliot et al., 2015; Julie et al., 2017). In this study, the classic sepsis model was established. The assessment of the successful establishment of the sepsis model was based on the degree of inflammation in the lung tissue (Lewis et al., 2016; Nwafor et al., 2019). In the model group, part of the alveolar wall collapsed, a large number of cells infiltrated and protein solutions exuded in the alveoli, and a large number of neutrophils aggregated in the interval. Observation of the distribution and number of red blood cells in the lung tissue shows that the LPS-induced lung inflammation during sepsis was evident, and the exfoliative cells in the LPS group indicated that the sepsis model had been successfully established.

From the results of the HE staining of the cerebral cortex and hippocampus, it can be found that in the LPS group, the cerebral cortex and hippocampal neuron cells were deeply stained, indicating a large amount of cell death. The loose arrangement of the cells in the hippocampus, which is related to cognitive function, indicated that inflammation had spread throughout the body and the brain. Moreover, the body weight of the rats in the LPS group decreased sharply within 14 days, and the mortality rate was as high as 50%. No deaths occurred in the TMP group. TMP has a protective effect on LPS-induced sepsis and can effectively improve the survival rate of rats.

A large number of studies have indicated that inflammatory cytokines, such as TNF-α, play a central role in Gram-negative bacteria-induced sepsis and endotoxic shock (Yiguan et al., 2014). Subsequent ELISA tests were performed on the serum to show the increased secretion of inflammatory cytokines in the serum of LPS-induced septic rats. The LPS-induced increases in the levels of the inflammatory cytokines TNF-α, IL-1β and IL-6 were decreased in the TMP group. Four hours after LPS administration, the behavior of the rats was affected, diarrhea symptoms appeared, the movements of the rats were slow and unstable, and obvious bleeding occurred in the eyes. In contrast, the rats in the TMP group had diarrhea and other symptoms at the beginning. However, the symptoms were basically controlled within 1 hour after the injection of TMP (Qiang et al., 2019; Dong et al., 2019). These results demonstrate that TMP can significantly inhibit the secretion of inflammatory cytokines to block inflammatory reactions. It has been reported that TMP may reduce the release of various inflammatory mediators and reduce the inflammatory response by inhibiting the activation of NF-κB p65 and activator protein 1 (AP-1) (Watanabe et al., 2005; Godfrey et al., 2011).

LPS-induced sepsis can damage the blood–brain barrier in the patient’s brain. In severe cases, cognitive impairment, delirium and even shock can occur. The effect of sepsis on brain function has a significant impact on the quality of life of patients with septic shock (Adrian et al., 2014). LPS-induced bacteremia destroys the blood–brain barrier of the brain through blood circulation and inflammatory responses (Gong et al., 2019). Since damage to the blood–brain barrier was difficult to observe, a tracer to detect BBB damage is needed. Evans Blue was chosen as a blood–brain barrier (BBB) tracer to determine the LPS-induced damage to the BBB. During the experiment, which tested the LPS dose and Evans Blue infusion duration, the infiltration of Evans Blue in the brains was investigated, and it was faint and not sufficiently sensitive for detecting lighter damage to the BBB due to its relatively large molecular weight. Thus, sodium fluorescein, with its small molecular weight, was used as a tracer; this tracer can more easily penetrate the BBB and is more sensitive to BBB damage. The results showed that TMP could protect the blood–brain barrier from LPS-induced sepsis. In addition, based on the IHC staining results, the tight junction proteins *claudin-1, claudin-5, occludin* and ZO-1, which are closely related to the blood–brain barrier of the brain, were all significantly reduced in the LPS group. The weakened tight junctions between the cells increased the degree of LPS-induced damage to the blood–brain barrier of the brain. The real-time PCR results showed that the levels of the tight junction genes including occludin, claudin-5 and *ZO-1* were reduced in the LPS group, and compared with the control group and the LPS+TMP group, the differences were significant, except for the difference in the *occludin* gene. This finding suggested that the destruction of the blood–brain barrier was directly related to the presence of tight junction proteins (Frolkis et al., 2009; Yi et al., 2017). TMP could protect against LPS-induced blood–brain barrier destruction and thus reduce the occurrence of delirium and restore cognition.

Studies have indicated that excessive NO is produced during sepsis, and the levels of nitrite and nitrate in the plasma of patients with sepsis are significantly increased (Adrian et al., 2014). Sepsis-related mediators, such as endotoxin and the pro-inflammatory cytokines IL-1β, IL-2, IL-6, TNF-α, and IFN-β, have been shown to induce high expression of iNOS (Frolkis et al., 2009; Godfrey et al., 2011; Dong et al., 2019). NO plays a vital role in LPS-induced death. Recent studies have shown that treatment with lysine base can inhibit the LPS-induced upregulation of the inducible nitric oxide synthase (iNOS) and cyclooxygenase 2 (COX-2) protein in RAW264.7 cells (Jingjing et al., 2011; Rui et al., 2014; Yi et al., 2017). Additional evidence has proven that the upregulation of nitric oxide synthase (NOS) has a devastating effect on the BBB in a variety of diseases. The study found that the NO content in the LPS group was significantly increased, and the expression of related proteins, such as iNOS and eNOS, was also significantly increased. The remarkable increase in the MDA content is an indicator of lipid oxidative damage, and the alteration of sphingolipid metabolism is a potential mechanism of the LPS-induced BBB destruction (Frolkis et al., 2009). Therefore, we speculate that the disruptive mechanism of LPS on the BBB of the brain may be related to nitric oxide synthase, and TMP may protect the blood–brain barrier and reduce the inflammatory response by inhibiting the expression of NOS (Jingjing et al., 2011).

## Conclusion

At present, TMP has already been developed for the treatment of a wide variety of ischemic cerebrovascular diseases (such as insufficient cerebral blood supply and cerebral thrombosis) *via* injection. This study found that TMP also exerted a promising protective effect on septic rats. The results showed that TMP treatment reduced weight loss, increased survival rates, mitigated lung and brain tissue injury, and decreased TNF-α, IL-1β, IL-6, MDA, and NO levels. In addition, TMP could protect the lamina and cerebral cortex, increase tight junction-related proteins, and inhibit nitrous oxide system indicators iNOS and eNOS. Above all, TMP ameliorates LPS-induced sepsis in rats by protecting the blood–brain barrier and suppressing inflammatory reactions and the nitrous oxide system. This study suggests a therapeutic approach for treating the deadly disease of sepsis in the clinic.

## Data Availability Statement

The raw data supporting the conclusions of this article will be made available by the authors, without undue reservation, to any qualified researcher.

## Ethics Statement

The animal study was reviewed and approved by The Animal Experimentation Ethics Committee of Ningbo First Hospital.

## Author Contributions

Y-JC: experiment, perform data collection. D-QX: Western blot and statistical analysis. Z-SH: writing-original draft, data curation. C-SH: conceptualization, formal analysis. L-JX: writing review and editing. MZ: validation, software and visualization; YC: overall design, control and responsibility. All authors contributed to the article and approved the submitted version.

## Funding

This research was supported by grants from the Ningbo Public Welfare Science and Technology Project (No. 2019C50079).

## Conflict of Interest

The authors declare that the research was conducted in the absence of any commercial or financial relationships that could be construed as a potential conflict of interest.

## Abbreviations

BBB, blood–brain barrier; eNOS, endothelial nitric oxide synthase; HE, hematoxylin–eosin; IHC, immunohistochemistry; iNOS, inducible nitric oxide synthase; LPS, lipopolysaccharide; MDA, malonaldehyde; NOS, nitric oxide synthase; NO nitric oxide; TMP, tetramethylpyrazine.
